# Intrinsic Ability of Adult Stem Cell in Skeletal Muscle: An Effective and Replenishable Resource to the Establishment of Pluripotent Stem Cells

**DOI:** 10.1155/2013/420164

**Published:** 2013-06-02

**Authors:** Shin Fujimaki, Masanao Machida, Ryo Hidaka, Makoto Asashima, Tohru Takemasa, Tomoko Kuwabara

**Affiliations:** ^1^Research Center for Stem Cell Engineering, National Institute of Advanced Industrial Science and Technology (AIST), Central 4, 1-1-4 Higashi, Tsukuba, Ibaraki 305-8562, Japan; ^2^Health and Sport Sciences, Graduate School of Comprehensive Human Sciences, University of Tsukuba, 1-1-1 Tennodai, Tsukuba, Ibaraki 305-8577, Japan

## Abstract

Adult stem cells play an essential role in mammalian organ maintenance and repair throughout adulthood since they ensure that organs retain their ability to regenerate. The choice of cell fate by adult stem cells for cellular proliferation, self-renewal, and differentiation into multiple lineages is critically important for the homeostasis and biological function of individual organs. Responses of stem cells to stress, injury, or environmental change are precisely regulated by intercellular and intracellular signaling networks, and these molecular events cooperatively define the ability of stem cell throughout life. Skeletal muscle tissue represents an abundant, accessible, and replenishable source of adult stem cells. Skeletal muscle contains myogenic satellite cells and muscle-derived stem cells that retain multipotent differentiation abilities. These stem cell populations have the capacity for long-term proliferation and high self-renewal. The molecular mechanisms associated with deficits in skeletal muscle and stem cell function have been extensively studied. Muscle-derived stem cells are an obvious, readily available cell resource that offers promise for cell-based therapy and various applications in the field of tissue engineering. This review describes the strategies commonly used to identify and functionally characterize adult stem cells, focusing especially on satellite cells, and discusses their potential applications.

## 1. Introduction

Stem cells are primordial cells common to all multicellular organisms and retain two distinctive properties: (1) the ability to self-renew through mitotic cell division and thus remain in an undifferentiated state and (2) the ability to differentiate into specific cell types [1, 2]. When a stem cell divides, each new cell has the potential either to remain a stem cell or become another type of cell with a more specialized function, such as a muscle cell, a blood cell, or a brain neuronal cell. Recent studies in the field of therapeutics suggest that stem cells will become a major focus in organ transplantation and replacement of lost tissue [3]. Stem cells can be categorized as totipotent, pluripotent, and multipotent, depending upon their differentiation potential [4, 5]. Totipotent stem cells arise through the fusion of an egg with a sperm and differentiate into embryonic and extraembryonic cell types. Pluripotent cells are the descendants of totipotent cells and can give rise to most of the tissues necessary for embryonic development.

 Embryonic stem (ES) cells are pluripotent, meaning that they can differentiate into all lineages of the primary three germ layers [6]: ectoderm, endoderm, and mesoderm, which are distinguished by their pluripotency and capability for indefinite self-renewal. Pluripotent stem cells originate as an inner cell mass within a blastocyst. The blastocyst contains three distinct areas: the trophoblast, which is the surrounding outer layer that later becomes the placenta, the blastocoel, which is a fluid-filled cavity within the blastocyst, and the inner cell mass, which becomes the embryo proper. ES cells can be created from cells taken from the inner cell mass. Because these cells represent such an early stage of development, they have the ability to become cells of any tissue type (except for the whole embryo itself), making them pluripotent. ES cells generate more than 220 cell types in the adult body, while adult stem cells are multipotent and can only produce a limited number of cell types [7].

 Induced pluripotent stem (iPS) cells are generated by reprogramming a differentiated somatic cell into a pluripotent ES cell using defined factors (Oct4/c-Myc/Klf4/Sox2) [8]. iPS cells appear to be an ideal substitute for ES cells, and many efforts have been made to improve methods of iPS cell generation and for understanding the reprogramming mechanism as well as the nature of iPS cells. The most important contribution of iPS cells to medicine may be the possibility of establishing personalized iPS cells for clinical applications without the need to harvest allogeneic human ES cells from embryos or deal with nuclear transfer [9]. The generation of patient-specific iPS cells for studies of genetic background and disease mechanisms is also useful approach for the screening of new drugs. Such customized iPS cells generated from patients can also be studied *in vitro* or *in vivo* as models for the pathogenesis of specific diseases [10]. One issue that hinders the clinical use of human ES cells is the lack of identical genetics between donor cells and recipients. This issue can be resolved using iPS cell. However, iPS cells generated from patients harboring genetic disorders cannot be applied for cell therapy, as iPS cell technology reprograms epigenetic, but not genetic, information in somatic nuclei. Several technologies have been developed for genome editing using disease-specific iPS cell lines [11, 12], and further elucidation of safety concerns and the mechanisms behind the differences in genetic background is required.

## 2. Adult Stem Cells

Pluripotency distinguishes ES cells from adult stem cells, which retain multipotency. Adult stem cells are undifferentiated cells contained throughout the body and divide to replenish dying cells and regenerate damaged tissue [13, 14]. They are also known as somatic stem cells. Adult stem cells have a close relationship with the surrounding tissue and the environment. Their niche is a specialized cellular microenvironment that provides them with the support needed for self-renewal [15, 16]. To ensure this, stem cells undergo two types of cell division. Symmetric division gives rise to two identical daughter cells both endured with stem cell properties. Asymmetric division produces only one stem cell and a progenitor cell with limited self-renewal potential [17, 18]. Progenitors can undergo several rounds of cell division before terminally differentiating into a mature cell.

 Bone marrow is the major source of adult stem cells. Hematopoietic stem cells can give rise to all blood cell types including both the myeloid (monocytes and macrophages, neutrophils, basophils, eosinophils, erythrocytes, megakaryocytes/platelets, and dendritic cells) and lymphoid (T cells, B cells, NK cells, and some dendritic cells). Bone marrow stromal stem cells are progenitors of skeletal tissue components such as bone, cartilage, hematopoiesis-supporting stroma, and adipocytes. Mesenchymal stem cells are multipotent stem cells that can differentiate into a variety of cell types *in vitro* or *in vivo*. Adult mesenchymal stem cells have been used in preclinical models for tissue engineering of bone, cartilage, muscle, marrow stroma, tendon, fat, and other connective tissues [19]. Although therapeutic applications are potentially acceptable, a rigorous understanding of mesenchymal stem cells requires a better definition of what such stem cells are, since currently there are no specific markers that can reliably discriminate between mesenchymal stem cells and others (e.g., fibroblasts).

 Muscle-derived stem cells have been shown to differentiate into myogenic, osteogenic, chondrogenic, adipogenic, and hematopoietic cells, similarly to mesenchymal stem cells [20]. In contrast, satellite cells that are committed myogenic stem cells are more restricted to skeletal muscle lineage [21]. Although satellite cells are considered to be the major stem cell source in skeletal muscle, many studies suggest that nonsatellite cells exhibit myogenic capacities [22–28]. Mesenchymal stem cells are able to display a skeletal muscle phenotype under appropriate conditions [29]; however, details of the mechanism of transdifferentiation remain elusive.

## 3. Muscle-Specific Adult Stem Cells—“Satellite Cell”—and the Development

Muscle tissue represents an abundant, accessible, and replenishable source of adult stem cells. Skeletal muscle accounts for a large proportion of total body weight, being over 30% for women and around 38% for men [30]. Myofibers are the basic cellular unit of skeletal muscle, and the syncytial myofiber is formed in the embryo through fusion of many myoblasts. Skeletal muscles are formed from the paraxial mesoderm, surrounding the neural tube, which separates into blocks known as somites. Dorsally, somites differentiate into epithelial dermomyotome, which then develops into myotome (the source of limb muscle), into dermatome (a specific region for nerve reception supplied by sensory neurons), and ventrally into mesenchymal sclerotome. The nuclei of myofibers originate from the myotomal somitic or lateral plate somatic mesoderm depending on the anatomical tissue location [31]. 

Cells within the dermomyotome exhibit specific expression patterns of Pax3 and Pax7. In the mouse embryo, Pax3(+) or Pax7(+) muscle progenitor cells in the dermomyotome enter the myotome in the central compartment of the somite from embryonic day 10.5 (E10.5). Initially, Pax3 and Pax7 are expressed ubiquitously throughout the dermomyotome. During embryonic development, Pax3(+)/Pax7(+) positive cells continually generate fetal myoblasts, which can be identified by their expression of Myf5 (the earliest marker of the myogenic lineage). Although the expression of Pax3 gradually decreases in the dorsomedial area and becomes predominant in the lateral dermomyotome, the expression of Pax7 becomes concentrated in the dorsomedial region of the dermomyotome [32]. Pax7(+) cells become lineage specific to muscle after E12.5 and occupy the sublaminal spaces of myofibers around E16.5, and expression of Pax7 is retained by the population of satellite cells (quiescent muscle progenitors; see below). Clonal analyses of satellite cells have suggested that satellite cells are heterogeneous with regard to their self-renewal abilities and the extent of the progeny they generate [33]. Pax7 is considered to be both a specific marker and essential for specification of the adult satellite cell pool [34]. 

**Figure 1:**
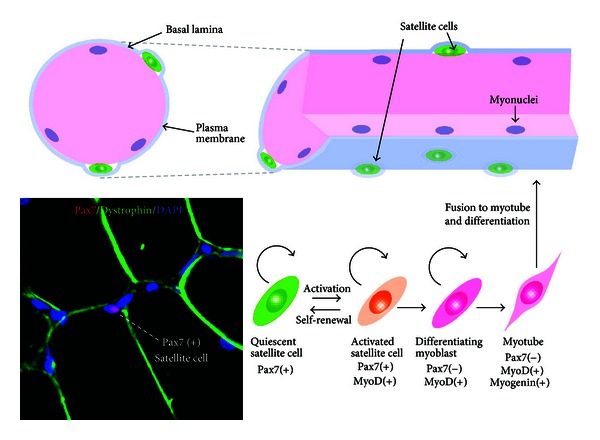
The self-renewal, activation, and differentiation of satellite cells in adult skeletal muscle. Satellite cells reside adjacent to the plasma membrane under the basal lamina on the surface of the myofiber. The nuclei of myofiber (myonuclei) are positioned at the periphery of the cell. Satellite cells are activated upon receiving various external stimuli and differentiate together with the upregulation of MyoD. Quiescent and activated satellite cells express a characteristic marker, Pax7. Immunohistochemical detection of Pax7 (red), dystrophin (green) and DAPI (blue) of adult rat skeletal muscle is shown (left bottom).

Satellite cells are muscle-specific stem cells identified by their direct attachment to the muscle fibers under the basal lamia (Figure 1) [35]. In adult muscle, satellite cells are in a quiescent state under normal conditions and represent 2.5–6% of all the nuclei of a muscle fiber. However, when activated by muscle injury, they can proliferate, undergo self-renewal, differentiate, and then generate a large number of new fibers within a few days [36]. The basal lamina contains several extracellular matrix proteins, such as collagen, laminin, fibronectin, and proteoglycans, and provides structural support by anchoring satellite cells. Skeletal muscle niches control the signaling of extracellular matrix materials, cell adhesion, and the behavior of satellite cells [37–39]. Identification of the molecular signals of stem cell niches is indispensable for understanding the action and function of satellite cells [39].

As an external stimulatory factor, exercise has a positive effect on the regulation of satellite cells. Several studies have indicated that the number of satellite cells increases after long-term or acute exercise training [40, 41]. This accretion of satellite cells in skeletal muscle is also evident in humans [42]. As a long-term effect of exercise, the trapezius muscle of trained power lifters contains 70% more satellite cells than that of control subjects [43]. The increased number of satellite cells after exercise gradually decreases during detraining, indicating that continuous exercise is required to maintain a rich satellite cell pool in skeletal muscle. Kurosaka et al. reported that the satellite cell pool following endurance training depends on the intensity rather than duration of exercise [44]. In various types of exercise, the effective method to increase and maintain satellite cell pool is investigated [45].

Since skeletal muscle is a flexibly changeable organ that can frequently increase or decrease in strength and mass and regenerate after injury, the stem cells included in the tissue can show dramatic changes in their fate depending on the circumstances of an individual's life. The balance that exists among self-renewal, differentiation, survival, activation, fusion, cell adhesion, and migration supported by these various extracellular signals is crucial for stem cell maintenance and muscle tissue homeostasis.

## 4. Molecular Mechanisms Regulating Satellite Cells

### 4.1. Paired Box Transcriptional Factor 7 (Pax7) and the MyoD Family

Satellite cells are a heterogeneous population and demonstrate at least two phases in skeletal muscle turnover: a mitotically quiescent state and an activated proliferative state (Figure 1). Both quiescent and activated satellite cells express a characteristic marker, Pax7 [46]. Quiescent satellite cells express Pax7 alone, whereas activated satellite cells coexpress Pax7, Myf5, and MyoD, which are key transcription factors for myogenic differentiation [46]. Although most Pax7(+)/MyoD(+) activated satellite cells proliferate and differentiate, accompanied by Pax7 downregulation, a small population of Pax7(+)/MyoD(−) satellite cells withdraws from the cell cycle and returns to a quiescent state [47]. Pax7-deficient satellite cells are gradually lost in skeletal muscle due to death or precocious differentiation [34, 48]. In particular, skeletal muscle mass and myofiber diameter are significantly reduced in Pax7^(−/−)^ mice [49].

 Activated satellite cells express Myf5 and MyoD, which are members of the MyoD family of basic helix-loop-helix (bHLH) transcription factors that play essential roles in regulating satellite cell differentiation and skeletal muscle development [36]. Myf5 is a target of the Pax7 transcription factor. Pax7 activates Myf5 expression through recruitment of a histone methyltransferase (HMT) complex, and the activator complex directly methylates histone H3 lysine 4 (H3K4) in the promoter region of Myf5 [50]. Kawabe et al. have reported that Pax7 is a specific substrate of coactivator-associated arginine methyltransferase 1 (Carm1), which is a protein arginine methyltransferase that methylates histone H3, and the methylation of Pax7 by Carm1 leads the recruitment of the HMT complex to the Myf5 locus [51]. Double-positive Pax7(+)/Myf5(+) satellite cells can upregulate MyoD, triggering proliferation [47, 52]. In this context, FoxO3 also contributes to MyoD upregulation by binding to the MyoD promoter region with Pax7 and recruiting RNA polymerase II [53]. The bHLH MyoD transcription factor initiates a differentiation program through association with E proteins (i.e., the E2A gene products, E12, E47, and HEB) by creating a heterodimer for the consensus E-box regulatory sequences on muscle-specific genes (myogenin, which is expressed at a more differentiated stage, Acta1, Lsp1, Mef2c, Tnnc2, Tnni2, Tnnt3, etc.) [54, 55]. For transcriptional activation, MyoD associates with HATs p300 and pCAF, which acetylate histones H3 and H4 [56, 57]. MyoD can also interact with the ATP-dependent chromatin remodeling factor SWI/SNF, leading to activation of the muscle-specific genes [58, 59]. Additionally, Rampalli et al. have demonstrated that MyoD collaborates with Mef2d to trigger the expression of the target myogenin gene [57]. Members of the Mef2 transcriptional regulator family are expressed in most tissues and play a critical role during myogenesis, with Mef2d being the skeletal muscle-specific isoform [60]. Mef2d accelerates the differentiation of skeletal muscle together with MyoD [61]. MyoD acts as master regulator of myogenesis to access and remodel chromatins and to induce the active transcription of muscle-specific genes. 

### 4.2. Notch Signaling Pathway

One candidate for regulating the quiescent state of satellite cells is the Notch signaling pathway, whose activity has been shown to regulate cell fate and proliferation in a variety of tissues [58, 59, 62, 63]. Binding of Notch receptors to their DSL ligands (Delta/jagged, Serrate, or Lag2) releases the Notch intracellular domain (NICD) [64]. NICD is translocated into the nucleus and binds to recombining binding protein-J*κ* (RBP-J*κ*) [65], which is a key mediator of Notch signaling and acts downstream of Notch receptors [66]. RBP-J*κ* inhibits transcription target genes by binding transcriptional corepressors in the absence of Notch signaling [67], while binding to NICD and displacing corepressors, leading to transcriptional activation in the presence of Notch [68].

Notch signaling regulates the proliferation and differentiation of activated muscle satellite cells [69]. Notch3 is expressed by quiescent satellite cells [70], and disruption of Notch3 results in loss of regulation of satellite cell proliferation [71]. Deletion of RBP-J*κ* in satellite cells specifically leads to their depletion through loss of their ability to regenerate after muscle injury [72, 73]. Whereas RBP-J*κ*-deficient satellite cells proliferate before fusion, most of them differentiate without the first division and fuse with adjacent myofibers, resulting in satellite cell depletion in muscles. Hes1, Hey1, and HeyL, which are downstream factors of Notch signaling, are highly expressed in quiescent satellite cells. Members of both the Hes and Hey families of bHLH repressors are induced by Notch. Hes1 or Hey1 inhibits the expression of MyoD via the formation of inactive Hes1/MyoD or Hey1/MyoD heterodimers [74, 75] and blocks the differentiation of satellite cells into myoblasts. Inhibition of myogenesis by Notch is critical for the expansion of the undifferentiated stem cell population, and expression of the target genes for Notch signaling contributes to regulation of the quiescence of satellite cells. 

### 4.3. Wingless-Type MMTV Integration Site Family (Wnt) Signaling Pathway

Wnt is a family of highly conserved secreted signaling molecules that play an essential role in the development and function of a variety of tissues. Wnt proteins typically bind to Frizzled receptors (Fzd) located in the plasma membrane [76]. The binding of Wnt and receptors activates *β*-catenin/TCF/LEF transcriptional complexes. *β*-Catenin, which is subunit of the cadherin protein complex and acts as an intracellular signal transducer, associates with its own degradation complex, resulting in its ubiquitin-dependent degradation [77]. However, when Wnt binds to Fzd receptors, *β*-catenin can translocate into the nucleus and bind members of the TCF and LEF family of transcription factors resulting in activation of target gene transcription [78]. This pathway through *β*-catenin is referred to as canonical Wnt signaling. In contrast, there are noncanonical Wnt signaling pathways that transmit signals through Rac/Rho activation, leading to cytoskeletal remodeling and induction of Jun target genes [79] and the PKC-calcium-dependent pathway [80].

**Figure 2:**
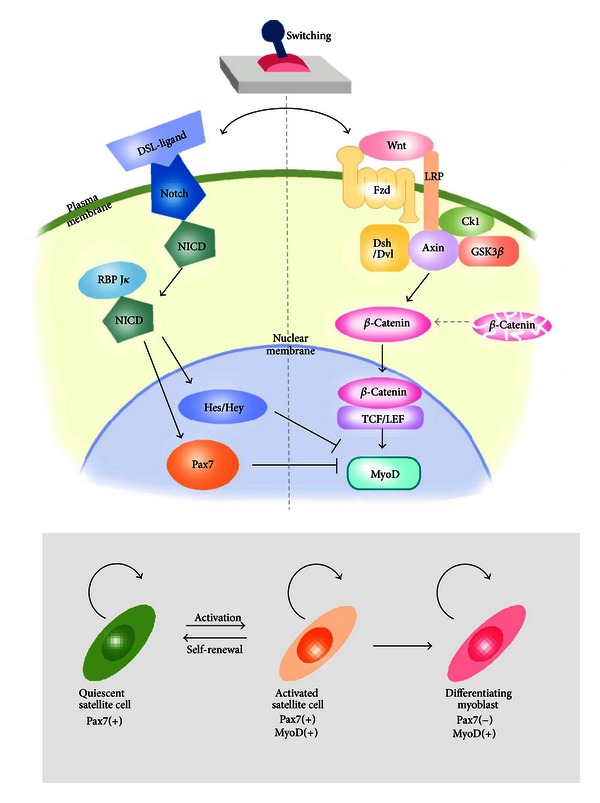
Regulatory switch between quiescent and activated states of satellite cells. Notch and Wnt signaling antagonize each other to define the state of satellite cells. Upon binding of DSL ligands (Delta) to Notch receptor, released Notch intracellular domain (NICD) translocates to the nucleus. After associating with RBP-J*κ*, the complex activates the transcription of target genes, such as Hes, Hey, and Pax7 to maintain satellite cells in quiescent state (left). When the “molecular switch” turns from the quiescent state to the activation state by Wnt proteins (right), the Wnt signal transduction activates *β*-catenin/TCF/LEF transcriptional complexes. The transcription complex triggers the expression of target MyoD gene and positively regulates myogenic differentiation.

It has been clarified that Wnt signaling is an important factor in the regulation of myogenesis, because of its influence on expression of the MyoD family. Wnt1 induces the expression of Myf5, whereas Wnt7a or Wnt6 preferentially activates MyoD in explant cultures of mouse paraxial mesoderm [81]. Wnt/*β*-catenin signaling has been shown to initiate the differentiation of satellite cells by replacing Notch signaling (Figure 2) [82]. Recently, Han et al. reported that R-spondin, which is an activator of the canonical Wnt signaling pathway leading to activation of *β*-catenin-dependent gene transcription, positively regulates myogenic differentiation [83]. Furthermore, Wnt7a regulates the self-renewal of satellite cells via noncanonical Wnt signaling [84]. These findings suggest that both the Wnt canonical and noncanonical signaling pathways play various roles in embryonic and postnatal skeletal muscle development including the cell fate choice of satellite cells.

### 4.4. Other Factors

Sox8 is a member of the Sox proteins, which play fundamental roles in developmental and differentiation processes in a variety of tissues [85], and Sox8-deficient mice show a reduction of overall body weight in postnatal life [86]. Schmidt et al. have demonstrated that Sox8 is confined to satellite cells and is downregulated during differentiation in parallel with downregulation of Sox9. Overexpression of Sox8 or Sox9 inhibits myotube formation and leads to an obvious reduction in the expressions of MyoD and myogenin [87], suggesting that Sox8 is a negative regulator of skeletal muscle differentiation and, like Pax7 or Notch/Delta, ensures the maintenance of a proper pool of satellite cells. 

 It has been shown that hypoxia influences the function of satellite cells. Gustafsson et al. have demonstrated that hypoxia maintains myoblasts in an undifferentiated state by activating the Notch signaling pathway [88]. Liu et al. have also shown that hypoxia promotes Pax7 expression in satellite cells through activation of the Notch signaling pathway [89], suggesting that oxygen levels in satellite cells play a role in maintaining a balance between quiescence and activation.

 Nitric oxide (NO) also regulates the state of satellite cells. Wozniak and Anderson have reported that the concentration of NO regulates the balance between quiescence and activation of satellite cells on myofibers. Satellite cells maintain a quiescent state in the presence of a normal concentration of NO, whereas injury activates satellite cells by altering the concentration of NO by stretching fibers or through dysfunction of NO synthase, eventually leading to the release of hepatocyte growth factor/scatter factor (HGF) from the extracellular compartment [90]. HGF acts as an activator of satellite cells and plays an essential role during the early phase of the repair process. NO-dependent satellite cell activation via HGF mediates many aspects of the inflammatory response, and further research is necessary to gain a better understanding of the processes of muscle healing and regeneration.

## 5. Effects of Aging and Myogenic Disorders on Satellite Cells

### 5.1. Satellite Cells and Aging

**Figure 3:**
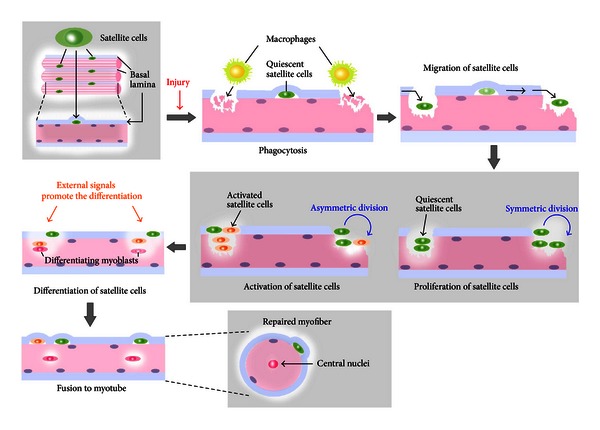
Satellite cell-mediated muscle regeneration upon injury. Damaged muscle fiber induces activation of quiescent satellite cells that reside between the plasma membrane and basal lamina. Macrophages digest myofiber debris at the damaged site (phagocytosis). Satellite cells migrate and proliferate through symmetric and asymmetric divisions. During the process, various external signals promote the differentiation of satellite cells and their fusions to myotube.

Regenerated myofibers contain central nucleus (Figure 3). It is important for satellite cells to maintain their regeneration potential in order to prevent any decrease of skeletal muscle mass during aging. The number of satellite cells declines in aged rodent and human skeletal muscles [91, 92]. The age-related decrease in the satellite cell population is one important cause of the sarcopenia, degenerative loss of muscle mass, strength, and frailty associated with aging. These observations suggest that maintenance of satellite cell number and function is important for allowing the advance of sarcopenia.

 Skeletal muscle has vigorous regeneration potential. Once skeletal muscle has been subjected to severe mechanical, chemical, or toxic stimulation, a proportion of myofibers are broken down and the resulting debris is subjected to phagocytosis by leukocytes such as neutrophils and macrophages (Figure 3). Satellite cells migrate to site of injury, where they rapidly proliferate and differentiate. Finally, skeletal muscle regeneration occurs through fusion of myoblasts into myofibers. Deletion of Pax7(+) cells disrupts this regeneration process [48], indicating the indispensable role of the Pax7 transcription factor in satellite cells. It has been reported that inhibition of leukocytes infiltration into injured skeletal muscle induces incomplete muscle regeneration and severe fibrosis [93]. Therefore, the environment surrounding satellite cells and the associated conditions also affect the regeneration process.

### 5.2. Changes in the Proliferation of Satellite Cells with Aging

It is well known that aging reduces the function of satellite cells, especially their proliferation potential. For instance, Schultz and Lipton have revealed that the number of satellite cells and the proliferation rate of isolated satellite cells decline with advancing age [94]. Carlson and Conboy recently indicated that the percentage of BrdU-positive satellite cells was reduced in aged mice relative to that in young mice under the same culture conditions [95]. In addition, many studies have indicated that aging severely affects the proliferation potential of satellite cells [96–98]. This decrease is associated with the muscle atrophy referred to as sarcopenia. The age-related decline in the proliferative ability of satellite cells impairs the regeneration potential of skeletal muscle.

The activation of satellite cells after muscle injury is controlled by Notch signaling, which is triggered by a rapid increase in the expression of Delta (a Notch ligand). In aged muscle, Delta fails to become upregulated after injury. Young muscles show significant upregulation of Delta upon injury, with Delta being expressed on the surface of satellite cells. Disruption of Notch signaling leads to a decrease in the number of satellite cells in aged skeletal muscle after injury [99]. Using heterochronic parabiosis, Conboy et al. have demonstrated that systemic factors in the blood of young mice can rejuvenate aged satellite cells and rescue the regeneration potential of aged mouse skeletal muscle [100]. Moreover, aged serum has been shown to reduce the percentage of Notch-positive satellite cells isolated from aged mice. BrdU-positive aged satellite cells can be increased by exposure to serum from young mice, and this upregulation is hampered when Notch signaling is inhibited. These results suggest that the dysfunction of Notch signaling induced in satellite cells by aged serum negatively affects their proliferation potential. Liu et al. have reported that satellite cell-specific constitutive Notch activation increases the number of satellite cells both *in vivo* and *in vitro* [89]. Additionally, Bjornson et al. have reported that satellite cell-specific Notch inhibition disrupts the regeneration of skeletal muscle and decreases the number of satellite cells after injury *in vivo* [72], suggesting that Notch inhibition reduces the satellite cell pool in skeletal muscle and that this is responsible for disruption of skeletal muscle regeneration.

Although the concentration of Notch is normal in aged muscle and aged satellite cells retain functional Notch receptors, Notch activation is impaired due to a lack of the Notch ligand, Delta [99, 101]. Since Delta is a regulator of satellite cell proliferation through Notch signaling, any alteration of Delta would modify the process of muscle repair. After muscle injury, satellite cells are activated and undergo explosive proliferation at the affected site (Figure 3). During this phase, activated Notch stimulates the proliferation and self-renewal of satellite cells, and these renewed satellite cells are the source of the subsequent explosive proliferation. Therefore, Notch inactivation by aging disrupts muscle regeneration due to a decline of the original satellite cell pool.

During proliferative activation of satellite cells induced by upregulation of Delta/Notch, transforming growth factor (TGF-*β*) signaling antagonizes this process. Carlson et al. reported that higher levels of TGF-*β* were expressed in aged than young satellite cell niches [102]. Increased expression of TGF-*β* during aging led to activation of phosphorylated SMAD (pSmad) and the subsequent signal transduction upregulated cyclin-dependent kinase (CDK) inhibitors, such as p15, p16, p21, and p27, via physical competition between Notch and pSmad3 on the promoters of CDK inhibitors. Increased expression of TGF-*β* with aging suppresses the proliferation potential of aged satellite cells, and a neutralizing antibody against TGF-*β* rescues this potential [97]. Both lack of Notch activation and upregulation of TGF-*β* synergistically inhibit satellite cell proliferation, resulting in deficient muscle repair and a decline of regeneration with aging. 

Another aging-related potential factor that acts as an extrinsic stimulus of satellite cells is Wnt. The downstream target of Wnt signaling, axis inhibition protein 2 (Axin2), is expressed at high levels in satellite cells derived from aged muscle, indicating a progressive increase of Wnt signaling during aging [96]. Injection of Wnt3a into young regenerating muscle after injury results in increased deposition of connective tissue, and exogenous Wnt induction also reduces cellular proliferation in young regenerating muscles that have a similar phenotype to aged muscle. These findings indicate that a low level of Wnt signaling is important for generating adequate levels of myogenic progenitors. Notch promotes proliferation and self-renewal of satellite cells as described above and also prevents their differentiation, that is, maintaining them in an undifferentiated state, by inhibiting Wnt signaling via induction of GSK-3*β* and degradation of *β*-catenin [103, 104]. Conversely, GSK-3*β* retains the ability to phosphorylate Notch-1 and Notch-2 [105, 106], suggesting that precise timing of the transition from Notch to Wnt and reciprocal control of the working stage from satellite cells to skeletal myoblast cells, respectively, is important for stem cell maintenance and the triggering of differentiation (Figure 2). The switch from stem cell proliferation to differentiation without loss of the original satellite cell pool is essential for the effective repair, regeneration, and aging of skeletal muscles. 

### 5.3. Muscle Fibrosis and Myogenic Disorders

After skeletal muscle injury, the repair process is initiated by release of growth factors and cytokines. Macrophages and fibroblasts that increase the production of extracellular matrix components migrate and proliferate. When normal regeneration occurs, these components are gradually degraded. Fibrous scar tissue is generated after muscle injury to fill the surface of the damaged area to facilitate regeneration. Although fibroblasts contribute to the repair response of tissues to injury by secreting extracellular matrix proteins such as collagen, fibrinogen, and fibronectin, continuous fibrosis is a pathological process in a variety of vital organs. Once fibrous scar tissue is overproduced in skeletal muscle, the muscle function becomes weaker. Pathophysiologic fibrosis due to accumulated extracellular matrix impairs muscle strength and can cause fibrotic diseases such as chronic myopathy and muscular dystrophy. In these conditions, fibrosis inhibits the diffusion of nutrients to myofibers [107]. Myostatin, also known as GDF-8, a member of the TGF-*β* family, is expressed in skeletal muscle and acts as an inhibitor of muscle growth by prohibiting the proliferation and differentiation of satellite cells, and thus deletion of myostatin causes muscle hypertrophy and hyperplasia [108]. Zhao et al. have reported that myostatin stimulates muscle fibroblast proliferation and expression of extracellular matrix proteins [109]. Persistent exposure to the inflammatory response increases the level of TGF–*β*1 [110], and this inhibits the activation of satellite cells and impairs myogenic differentiation [111]. 

Alexakis et al. showed that undifferentiated satellite cells express type I collagen, suggesting that satellite cells have the potential to adopt a fibroblastic phenotype [112]. Brack et al. reported that overexpression of Wnt3a resulted in abnormal extracellular matrix deposition and that aged mouse serum increased the population of nonmyogenic cells and fibronectin expression in satellite cells [113]. Since the aging-related fibrosis-converting phenomenon was diminished by induction of an inhibitor of Wnt3a, the Wnt signaling is also an important factor for induction of muscle fibrosis. On the other hand, quiescent satellite stem cells have shown to express the Wnt-receptor Fzd7, and Wnt7a significantly promotes the symmetric expansion of satellite stem cells (a 2-fold increase in the number of Pax7(+) satellite cells) via the planar cell polarity (PCP) pathway of noncanonical Wnt signaling [114]. Ectopic Wnt7a enhances muscle regeneration, suggesting an effect differing from that of Wnt3a, which causes fibrosis. These results suggest that there is cross-talk between Wnt7a and PCP and that Wnt3a/*β*-catenin signaling defines the fate of satellite stem cells, self-renewal, myogenesis, regeneration, and fibrosis during aging.

Macrophages, which are a vital component of the immune system, also play a role in muscle repair as they clear myofiber debris in regenerating and dystrophic muscle. Depletion or impairment of macrophages causes fibrogenesis in dystrophic muscle [93, 115]. Segawa et al. showed that reduction of macrophage infiltration using clodronate liposomes increased muscle fibrosis after injury, indicating that aging-induced dysfunction of immunity leads to fibrosis. Macrophages release the profibrotic molecules TGF-*β*, which activates fibroblasts to generate extracellular matrix. Excessive and persistent deposition of fibrinogen in the extracellular matrix inhibits the repair of myofibers, and fibrinogen accumulation is well correlated with advancing age [116]. Such deposition accelerates muscle inflammation and fibrosis. Further studies to reveal the mechanisms and molecules regulating inflammation and the promotion of fibrosis might provide an effective strategy for repair and healing of muscle injuries.

## 6. Transdifferentiation of Muscle-Derived Stem Cells

**Figure 4:**
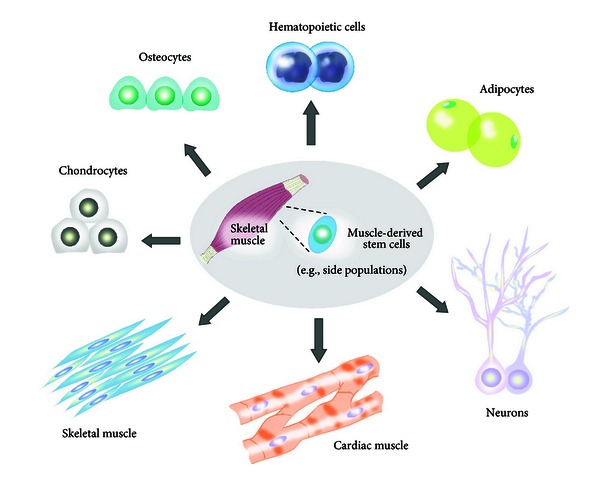
*Trans*-differentiation of muscle-derived stem cells. Possible *trans*-differentiation pathways of muscle-derived stem cells. Satellite cells retain a high intrinsic ability for* trans*-differentiation into multiple lineages (cardiac muscle, skeletal muscle, hematopoietic cells, adipocytes, neurons, chondrocytes, and osteocytes).

Satellite cells are considered to have specific unipotential for the myogenic lineage. While Pax7(+) satellite cells are committed stem cells for myogenic cells, stem-cell-like populations within skeletal muscle can differentiate through multiple pathways to form a variety of cell types and tissues (Figure 4). It is important to distinguish between defined satellite cells and muscle-derived stem cells (including side populations other than Pax7(+) cells). Muscle-derived stem cells that reside potentially as one of the origin of satellite cells [21] retain a high degree of flexibility and an intrinsic ability to exhibit multiple lineages.

### 6.1. Smooth Muscle

Unlike skeletal muscle, smooth muscle is an involuntary muscle found in the walls of blood vessels, the gastrointestinal tract, the bladder, or the uterus. Its structure differs from that of skeletal muscle in that it lacks visible cross-striations. Hwang et al. have reported that skeletal muscle-derived stem cells are able to differentiate into the smooth muscle lineage *in vitro* in response to vascular endothelial growth factor (VEGF) when cocultured with a feeder layer of smooth muscle cells. Two days of coculture of skeletal muscle-derived stem cells on a layer of smooth muscle cells converted them to alpha-smooth-muscle-actin- (*α*SMA-) positive cells [117]. Nolazco et al. have demonstrated that skeletal muscle-derived stem cells can transdifferentiate to the smooth muscle lineage when implanted into the rat corpora cavernosa and can correct aging-related erectile dysfunction, showing expression of *α*SMA, calponin 1, and smoothelin [117, 118]. Injection of skeletal muscle-derived cells into the urinary tract resulted in the formation of new myofibers [119]. Ho et al. have reported that skeletal muscle-derived stem cells grown on small-intestinal submucosa generated smooth-muscle cells expressing *α*SMA, calponin, and smoothelin [120]. These studies suggest that autologous transplantation of skeletal muscle-derived stem cells within a smooth muscle environment (niche) may be an effective approach for the treatment of smooth muscle injury.

### 6.2. Cardiac Muscle

Cardiac muscle is one of three major types of muscles (skeletal, cardiac, and smooth muscles) and is an involuntary type of striated muscle. The cardiomyocytes that compose cardiac muscle have a large number of mitochondria, myoglobin (an oxygen-storing pigment) for providing nutrients and oxygen, and show anaerobic metabolism. In contrast to skeletal muscle cells, cardiomyocytes are ischemia intolerant. Since the heart lacks functional repair mechanisms, a number of studies have investigated muscle-derived stem cells (skeletal muscle) for cardiac repair [121, 122]. 

Taylor et al. reported that autologous implantation of skeletal myoblasts in cryoinjured rabbit hearts improved systolic and diastolic function [121]. Skeletal myoblasts express N-cadherin and connexin43, which are major components of the gap junction, but these are downregulated as the cells terminally differentiate. Cardiomyocytes are electromechanically coupled by cell-cell junctions, the intercalated disks, including gap junctions for electrical communication between the cells [123]. The *β*-adrenergic agonist isoproterenol increases the synchronized beating rate between muscle-derived stem cell (skeletal muscle) grafts and cardiomyocytes, and heptanal (a gap junction blocker) inhibits contraction and restricts individual cardiomyocytes to their intrinsic pacemaker frequency [124]. Tamaki et al. have reported that freshly isolated CD34(+)/CD45(−) cells derived from skeletal muscle were converted to cardiomyocytes in a myocardial infarction model (left ventricle, LV) and that transplantation resulted in significant functional recovery (LV function: percentage of fractional shortening, regional wall motion score, ejection fraction, etc.). In a phase I clinical study, Herreros et al. have examined autologous skeletal myoblast transplantation in 12 patients with old myocardial infarction (MI) undergoing coronary artery bypass surgery. Okada et al. have also reported that transplantation of human muscle-derived stem cells (skeletal muscle biopsy samples from three human subjects) into an acute MI model in NOD/SCID mice significantly improved survival and engraftment, stimulated angiogenesis, and improved LV function. Transplantation of muscle-derived stem cells (skeletal muscle) was more effective than that of committed skeletal myoblasts, suggesting that high intrinsic and feasible regenerative ability of muscle-derived stem cells is important for MI repair [125]. These results suggest the usefulness of autologous cellular cardiomyoplasty using muscle-derived stem cells from skeletal muscle.

### 6.3. Osteogenic Lineage

Myoblasts derived from skeletal muscle treated with bone morphogenetic proteins (BMPs) or adipogenic-inducing agents differentiate into osteocytes or adipocytes [126, 127]. Katagiri et al. demonstrated that BMP-2 and TGF–*β*1 inhibited myotube formation in C2C12 cells (myoblast cell line originating from muscular tissue satellite cells) and downregulated the expression of MyoD and myogenin [128]. C2C12 cells induced by BMP-2 alone differentiated into osteoblast lineage. Chalaux et al. have shown that JunB contributes to the inhibition of myogenesis by BMP-2 and TGF–*β*1. Namiki et al. further demonstrated that BMP-2-dependent osteoblast differentiation was transduced via BMPR-IA (BMP receptor), and Akiyama et al. also showed that the transduced expression of BMPR-IB exhibited osteoblast-specific phenotypes in C2C12 cells [129, 130]. Lee et al. also reported that muscle-derived stem cells became hypertrophic and expressed the bone-specific marker osteocalcin/BGLAP under *in vitro* culture conditions with BMP-2 [131]. However, when BMP-2 was absent, myogenesis with myotube formation occurred. Kawasaki et al. demonstrated that BMP-2 inhibited myotube formation using primary cells extracted from human muscle tissue (pectoralis major, gluteus maximus, and adductor magnus) [132]. Yamamoto et al. demonstrated that Smad1 and Smad5, which mediate BMP signaling, were involved in the process of myogenic inhibition and the induction step of osteoblast differentiation [133]. 

Transplantation of muscle-derived stem cells has been attempted for repair of defects in bone. Muscle-derived stem cells transduced with viral vectors encoding BMP induced bone formation and improved bone healing [134, 135]. The transplanted muscle-derived stem cells responded to the secreted BMP-2 in an autocrine manner. Musgrave et al. reported that primary cells derived from human skeletal muscle could be used to produce bone in SCID mice [136]. Injection of the human cell expressing BMP-2 into the hind-limb muscle of the SCID mice caused ectopic osteogenesis within a few weeks. When these cell grafts are employed for orthopedic applications, extracellular matrix scaffolds support the process. Usas et al. demonstrated that delivery of BMP4-secreting muscle-derived stem cells was able to induce osteogenesis in mice if used with collagen gel, fibrin sealant, and gelatin sponge carriers and showed that a gel scaffold was more suitable for bone formation than sponge material [137]. Up to now, orthopedic treatment involving engraftment of allografts supplemented with demineralized bone matrix or vascularized bone grafts has been limited because of the reduced osteogenic capacity of the donor bone-forming cells. Muscle-derived stem-cell-based regenerative approaches for bone formation are potentially attractive as they exploit the intrinsic ability of muscle stem cells as a replenishable cell source for autologous transplantation.

### 6.4. Adipogenic Lineage

In aged mice, muscle shows a decrease of regenerative and proliferative capacity, with consequent loss of muscle mass, and myoblasts express higher levels of adipocyte lineage genes (adipose-specific FABP, C/EBP*α*, and PPAR*γ*), although a fully differentiated adipocyte phenotype is not achieved. Muscle-derived stem cells exposed to adipogenic inducers *in vitro* differentiate into adipocytes with a characteristic polygonal morphology and lipid-filled vacuoles in their cytoplasmic fractions. Replacement of muscle tissues by adipose tissues has been demonstrated in mutant mice (MyoD^(−/−)^ : Myf5^(−/−)^), and adipogenic potential has been examined in not only murine [138] but also human studies [139]. The process of adipogenesis is enhanced by the insulin sensitizer rosiglitazone [140]. Reagents (long-chain fatty acids and/or thiazolidinediones) that activate the peroxisome proliferator-activated-receptor- (PPAR-) *γ* could cause upregulation of genes involved in fatty acid uptake, storage, and metabolism in skeletal muscle tissues [141]. PPAR*γ* is required for insulin responsiveness of fat cells, and the expression levels of (age-related) adipogenic transcription factors determine the size of fat cells, and their capacities to store lipid and insulin respond to insulin. Elevated PPAR*γ* expression in skeletal muscle increases insulin sensitivity [142], and knockout of PPAR*γ* in skeletal muscle using a Cre-loxP system results in glucose intolerance and insulin resistance [143]. 

Aguiari et al. have reported that muscle-derived stem cells differentiate into adipogenic lineage upon exposure to high levels of glucoses, which in turn induces reactive oxygen species (ROS) and activation of the downstream effector kinase, PKC*β* [144]. ROS are byproducts of normal cellular metabolism that can cause cellular damage through oxidation of lipids, and PKC*β* plays a role in signaling that connects ROS with mitochondrial targets. These data suggest that *trans*-differentiation from muscle-derived stem cells to adipose lineage reflects oxidative stress, and thus converted cells are considered to be damaging cell populations in the age-related organ dysfunction induced by ROS. Elevated superoxide accelerates age-associated muscle atrophy through mitochondrial dysfunction, causing irreversible cell injury and death, and these changes to muscle tissue are responsible for the pathogenesis of sarcopenia [145, 146]. Oxygen concentration also modulates the *trans*-differentiation of muscle-derived stem cells to adipogenic lineages [147]. Further identification of the signals, cellular stage and specification, and molecular mechanism that underlie the process of *trans*-differentiation into adipose lineage may provide a deeper understanding of the intrinsic abilities of muscle-derived stem cells in nature and also the pathogenesis of sarcopenia. Muscle-derived stem cells retain the capacity to enter either a static myogenic differentiation pathway or a mesenchymal cell-like widely varied alternative differentiation pathway. 

### 6.5. Neuronal Lineage

The function of skeletal muscle is intimately dependent on the central and peripheral nervous systems. Functional linkage between skeletal muscle and the nervous system at neuromuscular junction is necessary for the normal function of various organs. Although a number of methods have been explored, muscle-derived stem cells can be induced to adopt neuronal lineages [148–152]. Arsic et al. reported that muscle-derived stem cells (skeletal muscle) began to express N-CAM, *β*-tubulin III, and GFAP [153]. Alessandri et al. isolated muscle-derived stem cells from human brachioradialis muscle of 12 patients and demonstrated that these cells differentiated into skeletal muscle fibers with a smooth-muscle cell phenotype (expression of smooth-muscle actin) and a neuronal phenotype (expression of *β*-tubulin III, GFAP, S100) *in vitro* [148]. Vourc'h et al. also showed that a particular population of CD34(+)/CD45(−)/CD90(+) cells isolated from adult skeletal muscle by rapid cell sorting gave rise to a significant number of cells entering the neuronal lineage [154]. Kwon et al. reported that valproic acid (VA), a histone deacetylase inhibitor used in the treatment of epilepsy, bipolar disorder, led to differentiation of muscle-derived stem cells toward neuronal lineages. Recently, Kang et al. demonstrated that fibroblast growth factor (FGF) and ethosuximide, which is used clinically to treat absence seizures in humans, induced neuronal differentiation of muscle-derived stem cells by showing immunohistochemically positive cells for TuJ1, NeuN, and neurofilaments M and H [150]. Further studies to reveal the mechanistic background of the conversion into the neuronal lineage may reveal further potential applications of muscle-derived stem cells, which can easily regenerate and consequently be rapidly expanded *ex vivo*, for repair of neuronal injuries and treatment of neuronal disorders.

### 6.6. Hematopoietic Lineage

Muscle-derived stem cells also have the ability to differentiate into hematopoietic cell lineages. Bellayr et al. showed that muscle-derived stem cells differentiated into hepatocyte lineage with liver regeneration ability [155]. Stem cell populations within skeletal muscle were capable of not only muscle regeneration but also hematopoietic engraftment (bipotent lineage potential) [22, 156–158]. Farace et al. reported that muscle-derived stem cells showed hematopoietic activity more than 10-fold than that of bone marrow giving rise to myeloid, T, B, and natural killer cells [159]. Regarding the origin of the muscle-derived stem cell showing myogenic and hematopoietic lineages, there are possibilities that hematopoietic stem cells are the plastically circulating stem cells or that primitive pluripotent stem cells in adult tissues [160, 161], for example, very small embryonic-like cells (VSEL), contribute to give rise to multiple lineages [162, 163]. If the yield and sensitivity of blood-forming human muscle-derived stem cells could be improved further, skeletal muscle would become a useful alternative cell source for autologous bone marrow transplantation, especially for patients with aplastic anemia. 

### 6.7. Chondrogenic Lineage

Muscle-derived stem cells have been shown to have the capacity to generate cartilage. Articular cartilage has a limited healing capacity because of its poor vascular supply. Transforming growth factor *β* (TGF-*β*), BMPs, insulin-like growth factor 1 (IGF-1), and basic FGF can improve chondrocyte proliferation and extracellular matrix synthesis. Subpopulation of myogenic progenitor cells, accumulating in the callus tissue of bone fractures, has been shown to express the cartilage marker collagen II in a mouse model of fracture healing [164]. L6 myoblasts and C2C12 myoblasts were able to differentiate into chondrocytes when treated with demineralized matrix or BMP-2 [165]. These data suggest that muscle progenitor cells have intrinsic ability to undergo chondrogenic differentiation in specific circumstances and that this process may be important for cartilage regeneration [166]. 

Transplantation of stem cell populations is an attractive approach for more efficient repair of articular cartilage defects. Adachi et al. have reported that skeletal muscle-derived stem cells promote cartilage repair when used with collagen gel [167]. Huard's group has reported monolayers of BMP-4-expressing muscle-derived stem cells from type II collagen-positive colonies, suggesting that chondrogenesis and TGF-*β* further promote *trans*-differentiation [168, 169]. The same group has also demonstrated that intracapsular injection of muscle-derived stem cells expressing BMP-4 and soluble Flt-1 is effective for repairing articular cartilage after induction of osteoarthritis and that platelet-rich plasma can promote collagen synthesis, thus increasing the therapeutic potential through inhibition of chondrocyte apoptosis [170]. The combination of IGF-1 and TGF–*β*1 (IGF-1/TGF–*β*1), TGF–*β*2/BMP-7, TGF–*β*2/BMP-6, TGF–*β*2/BMP-2, and TGF–*β*2/IGF-1 enhanced the conversion of muscle-derived stem cells into chondrogenic lineage [171, 172]. Cairns et al. have reported that Nkx3.2 plays a central role in the chondrogenic differentiation during the step at which Sox9 promotes chondrogenesis and inhibits myogenesis [173]. Further clarification of the intracellular molecular mechanisms, effective scaffolds, activating signaling molecules, and growth factors operating during the* trans*-differentiation of muscle derived-stem cells or myoblasts into the chondrogenic lineage may accelerate their utility for tissue engineering aimed at cartilage repair.

### 6.8. Angiogenic Lineage

Restoration of the vascular network for the exchange of oxygen, carbon dioxide, nutrients, and waste products is essential for muscle regeneration. Therefore, for muscle regeneration, every muscle construct is connected to a vascular system, and angiogenesis is controlled in a spatiotemporally coordinated manner. Angiogenesis is controlled largely by hypoxia-driven transcriptional upregulation and secretion of vascular endothelial growth factor (VEGF), and the expression of VEGF, angiopoietin 1/2, monocyte-chemoattractant-protein- (MCP-) 1, and their receptors (VEGFR, VEGFR2, etc.) strongly increases after injury [174]. Skeletal muscle-derived cells express the vascular endothelial markers VE-cadherin, VEGF-R2 (VEGF receptor), and smooth muscle *α*-actin [175, 176]. Bryan et al. have reported that VEGF expression is mediated by MyoD, since the VEGF promoter contains three tandem CANNTG consensus MyoD binding sites [177]. Furthermore, VEGF-null ES cells exhibit impaired myogenesis compared with wild-type ES cells, suggesting that VEGF retains an important role in the skeletal lineage differentiation program. Interestingly, undifferentiated C2C12 myoblast cells express VEGF-R1 and VEGF-R2 at low levels, and the expression levels of the VEGF receptors are upregulated upon differentiation of skeletal muscle [177]. These data suggest that myotube hypertrophy is coupled with both VEGF stimulation and also receptor activation and that the myogenic differentiation program is tightly regulated together with angiogenesis in an autocrine manner. Furthermore, there is a possibility that cancer patients undergoing long-term anti-VEGF therapy may suffer impairment of muscle regeneration as a side effect. Further research might shed further light on the possible clinical application of skeletal muscle-derived stem cells for reconstructive vascularization.

The adult skeletal muscle compartment is a complex organ because of the diversity of its lineages and its intrinsic potential for the *trans*-differentiation of muscle-derived stem cells. Muscle-derived stem cells can also act as mediators by releasing angiotrophic, neurotrophic, chondrogenic, hematopoietic, adipogenic, osteogenic, and other growth factors, thus supporting and further activating endogenous mechanisms for regeneration. Regenerative therapies using muscle-derived stem cells hold promise for the treatment of various diseases/disorders and also for injury repair. On the other hand, recent studies have indicated that somatic cells can be reprogrammed into iPS cells for clinical application. Skeletal muscle-derived cells themselves retain high utility as a cell resource for reconstructive tissue engineering but are also a useful and efficient source for the establishment of iPS cells because of their highly flexible intrinsic abilities and replenishable properties. 

## 7. iPS Cells from Skeletal Muscle Cells

Muscle satellite cells are responsible for the robust regeneration capacity of adult skeletal muscle. Satellite cells derived from skeletal muscle are capable of repopulating the stem cell pool, implying that they retain direct potential for the therapy of degenerative muscle disorders. After exercise or muscle injury, large numbers of muscle fibers are newly generated within a short period. Skeletal muscle cells are attractive as a source of iPS cells since they can be replenished easily. Although the efficiency of the reprogramming of somatic cells, such as fibroblast, into iPS cells is generally quite low, adult neural stem cells or hematopoietic stem cell cells reprogram efficiently [178, 179]. Therefore, muscle-derived stem cells or (myogenic) satellite cells are thought to be feasible cell source for iPS generation.

Polo et al. have reported that freshly isolated skeletal muscle precursors can be reprogramed to iPS cells effectively [180]. Established iPS lines gave rise to differentiated teratomas, and all tested lines supported the development of chimeric animals after blastocyst injection. Tan et al. have also demonstrated that skeletal muscle precursor cells (CD45(−)/Mac1(−)/Sca1(−)/*β*1-integrin(+)/CXCR4(+) satellite cells) and Sca1(+) mesenchymal progenitors from skeletal muscle can be reprogrammed into iPS cells with greater efficiency than differentiated CXCR4(−) cells using clonal assays and a second-generation inducible reprogramming system [181]. This indicates that pluripotency can be induced more efficiently in stem cells than in their more differentiated progeny. Furthermore, Watanabe et al. have reported that iPS cells can be generated from fully committed myogenic cells (MyoD-positive primary myoblasts) by retroviral transfer of four factors (Oct4/c-Myc/Klf4/Sox2) [182]. The muscle-derived iPS cells exhibited characteristics similar to those of ES cells and formed embryoid bodies and teratomas and contributed to the production of chimeric mice and their offspring, demonstrating their potential to develop into all three germ layers as well as into germ cells. 

Importantly, continuous expression of the MyoD gene inhibited the step of reprogramming into iPS cells, since MyoD expression alone can program many nonmuscle cells to undergo differentiation into the myogenic lineage [183]. The efficiency of iPS cell generation was much higher when muscle from MyoD^(−/−)^ mice was used for iPS production. Since Oct4 prevents the expression of MyoD, ectopic expression of Oct4 in myoblasts immediately shuts down MyoD gene expression. Lang et al. have reported that Oct4 also suppresses the expression of Pax7 by binding to the regulatory region of the Pax7 gene [184]. Moreover, Oct4 is able to bind to the upstream regulatory region of Cdx2 and Cldn4, genes that are specific for trophectoderm, to repress their expression [185]. Oct4 binds at the transcriptionally inactive Myf5 locus in ES cells [186], but recent ChIP-Seq analysis has shown that Oct4 does not bind to MyoD or the Pax7 locus in ES cells [187]. These data suggest that other transcription factor(s) containing a POU domain or cofactor protein(s) of Oct4 may negatively regulate MyoD expression indirectly. A consistent notion is that Oct4 is required for the initial reprogramming step in the induction of pluripotent stem cells from muscle cells. Although early passage iPS cells tend to retain an epigenetic memory of their somatic cell of origin, which reinforces biased commitment potential [188], iPS cells generated from different cell types in skeletal muscle show equal pluripotency. Adult stem cells reprogram more efficiently than terminally differentiated cells. Further studies aimed at identifying the molecular mechanism that prevents reprogramming from muscle tissues would facilitate effective generation of iPS cells for disease modeling, drug discovery, and cell therapy.
